# A Case of Successful Explantation of an Infected Fenestrated Aortic Endograft Using a Composite Xeno/Biosynthetic *In Situ* Reconstruction

**DOI:** 10.1016/j.ejvsvf.2025.01.001

**Published:** 2025-01-13

**Authors:** Tania Panettella, Maani Hakimi, Juan Antonio Celi de la Torre

**Affiliations:** a Department of Hand and Plastic Surgery, LUKS | Luzerner Kantonsspital, Universitäres Lehr- und Forschungsspital, Lucerne, Switzerland; b Department of Vascular Surgery, LUKS | Luzerner Kantonsspital, Universitäres Lehr- und Forschungsspital, Lucerne, Switzerland

**Keywords:** Aorta, Endovascular aneurysm repair, FEVAR, Case report, Vascular graft and endograft infection

## Abstract

**Introduction:**

Graft infections after open or endovascular repair can be devastating, and their treatment is always challenging. For thoraco-abdominal and abdominal aortic aneurysms, fenestrated and branched endografts are used increasingly. Because of the involved materials and anatomy, infective complications can be even more complex.

**Report:**

One year after double fenestrated endovascular endorepair for a type Ia endoleak after standard endovascular repair, a 77 year old patient developed clinical signs for sepsis at an external clinic. As his clinical situation deteriorated, he was then referred to the centre, where an infection focus search revealed a *Staphylococcus aureus* bacteraemia, and computed tomography (CT), and fludeoxyglucose positron emission tomography CT showed signs of endograft infection. Trestment by endograft explantation followed, and *in situ* reconstruction with a composite xeno/biosynthetic graft was performed. Through a median laparotomy, endograft explantation as well as *in situ* reconstruction were technically successful, and sepsis control was achieved under concomitant anti-infective therapy. After a 48 day hospital stay (22 days in the intensive care unit), the patient was discharged to a rehabilitation clinic. After three months of uneventful follow up, precision dual antibiotic therapy with ciprofloxacin and rifampicin was stopped. Four year follow up confirmed freedom from infection and a properly functioning aortic reconstruction.

**Discussion:**

After fenestrated stent graft procedures, successful late conversion is challenging and is known to correlate with high morbidity and mortality. The present case confirms the feasibility of this approach, even in patients with sepsis, with good results.

## Highlights

• Fenestrated endograft infection can be devastating.
• Late open conversion correlates with high morbidity and mortality rates.
• Xeno/biosynthetic *in situ* reconstruction is feasible but challenging.
• State of the art peri-operative care is crucial for patient survival.

## Introduction

Branched or fenestrated endovascular aortic aneurysm repair (FB-EVAR) is increasingly used for complex abdominal and thoraco-abdominal aneurysm repair. A rare but feared complication is endograft infection (EI), which is associated with high mortality.^1^, ^2^, ^3^ EI after FB-EVAR is, because of the extended number of device modules and the involved organ vessels, challenging and associated with lethal outcomes.^4^ With rising FB-EVAR numbers, the incidence of EI after FB-EVAR may increase as well.^5^ Despite the existence of EI case reports after FB-EVAR^4^^,^^6^^,^^7^ and clinical recommendations and guidelines^5^^,^^8^ for EI, the therapeutic approach must nevertheless be evaluated on a case by case basis.

In the present case of a 77 year old patient, an infected, double fenestrated FB-EVAR was successfully explanted. *In situ* reconstruction was performed using a composite bovine pericardium/biosynthetic Y graft. This case report was prepared in accordance with the SCARE and PROCESS criteria.^9^^,^^10^

## Report

A 77 year old man with a medical history of coronary disease, chronic obstructive pulmonary disease, and radiation therapy for micro-invasive squamous cell carcinoma of the vocal folds underwent EVAR in 2013 for an asymptomatic infrarenal aortic aneurysm 56 mm in diameter (Zenith Flex; main body 28–96 mm, contralateral iliac leg 16–90 mm Spiral Z, ipsilateral iliac leg 16–36 mm LP; Cook Medical, Bloomington, IN, USA) (Table 1).

**Table 1:** Timeline of endovascular procedures.

Date	Indication	Procedure/Material
2013	Asymptomatic AAA Ø 56 mm	EVAR (Zenith Flex. Main body 28–96, contralateral iliac leg 16–90 Spiral Z, ipsilateral iliac leg 16–36 LP)
05/2015	Thrombosis of the right prosthetic leg	Distal extension (Advanta V12, 16-41)
03/2018	Type Ia endoleak	Endorepair w/proximal extension cuff (TREO 28-28-40)
09/2018	Endoleak persistence and aneurysm growth Ø 65 mm	FEVAR (Anaconda, Advanta. Main body 32–65, contralateral leg 12-12-120, ipsilateral 12-15-110. Bridging stents: Advanta 7–22 r/l)

AAA = abdominal aortic aneurysm; EVAR = endovascular aneurysm repair.

All dimensions are in millimeters.

In 2015, due to a partial thrombosis at the wall of the right prosthetic limb, endolining by distal extension was performed with a covered stent (Advanta V12, 16–41 mm, Getinge AB, Göteborg, Sweden).

In 2018, endorepair with a proximal extension cuff (TREO, 28-28-40 mm, Terumo Aortic, FDBA Bolton Medical Inc., Inchinnan, UK) for a secondary type Ia endoleak was necessary due to progressive disease and loss of neck length. Because of persistent endoleak and aneurysm growth up to 65 mm, proximal extension with a custom made, double fenestrated FEVAR (Anaconda; main body 32-65 mm, contralateral leg 12-12-120 mm, ipsilateral 12-15-110 mm, Terumo Aortic, FDBA Bolton Medical Inc.; Advanta bridging stents, 7–22 r/l, Getinge AB, Göteborg, Sweden) was performed six months later.

One year after endovascular completion, an open surgical thrombectomy of the right popliteal artery for embolic occlusion was required. Furthermore, an acute coronary syndrome required several percutaneous interventions.

**Figure 1:**
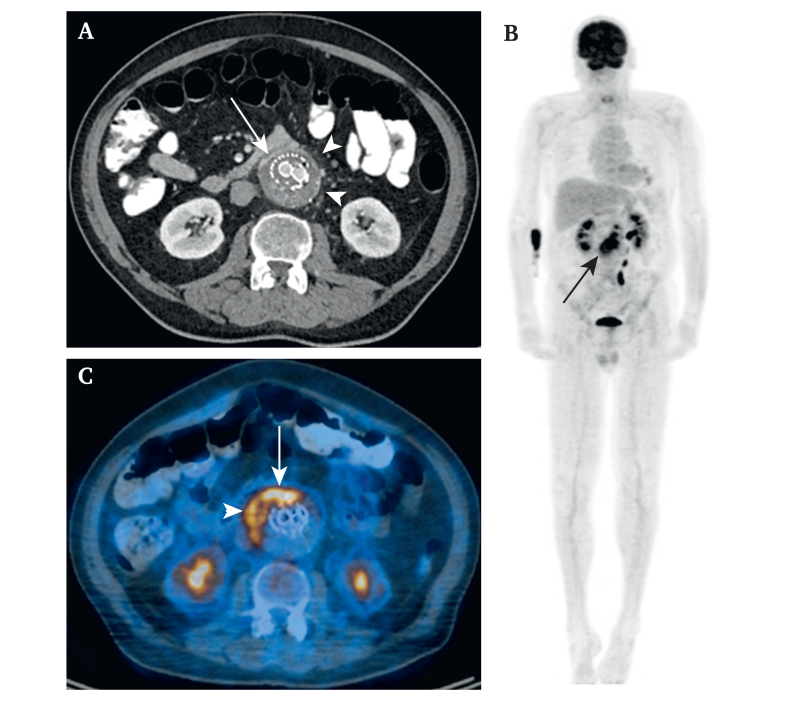
Multislice computed tomography angiography. (A) Transverse plane: peri-aortic enhancement (arrow), peri-aortic fat stranding (arrow heads). (B) Maximum intensity projection and (C) fused axial positron emission tomography CT images: intense, non-homogeneous fludeoxyglucose uptake of the stent graft (arrow in B and C) and of the thrombotic material in the aneurysm sac (arrow in B, arrowhead in C). Credits: Department of Nuclear Medicine and Radiology, Lucerne Cantonal Hospital.

**Figure 2:**
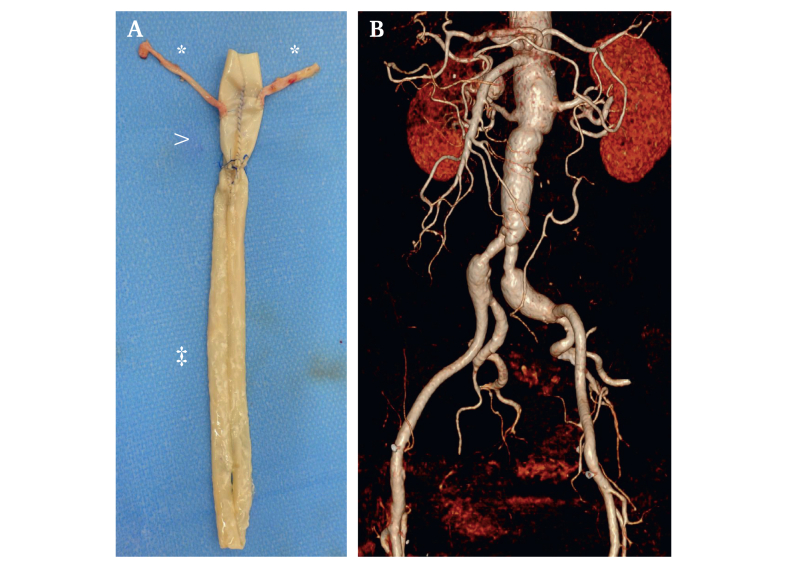
(A) Completed composite graft. ∗Venous bypasses to right and left renal arteries (RRA and LRA); main body: bovine pericardial surgeon made tube graft; ^‡^biosynthetic tubes (Omniflow, LeMaitre Vascular Inc. [FDBA Bio Nova International Pty Ltd], Burlington, MA, USA). (B) 3D volume rendering of the reconstruction two years after surgery; unchanged dilatation of the biosynthetic to common iliac artery reconstruction on both sides.

In the further course, during rehabilitation, there was a sudden deterioration in the patient's condition. Signs of incipient sepsis, including leucocytosis (18.7 x10^9^/L) and an increase in C-reactive protein (CRP) (303 mg/L) despite intravenous antibiotic therapy, led to a re-admission. CT (CT) and fludeoxyglucose positron emission tomography CT showed EI (Fig. 1). Clinical evaluation showed abdominal discomfort and fever above 38.0°C. Blood cultures revealed a *Staphylococcus aureus* bacteraemia, thus fulfilling five minor MAGIC criteria indicating EI.^8^ Emergency explantation of the infected endograft was indicated. The neo-aortic reconstruction was designed as a xeno/biosynthetic Y graft (Fig. 2A): the main body consisted of a hand sewn bovine pericardial tube graft (XenoSure, Biological Patch, LeMaitre Vascular Inc., Burlington, MA, USA), extended with two single Omniflow II vascular prosthesis tubes (LeMaitre Vascular Inc. [FDBA Bio Nova International Pty Ltd], Burlington, MA, USA). Two vein segments (left and right great saphenous veins) were inserted laterally into the main body anticipating the need for renal artery replacement due to dissection after endograft removal.

After midline laparotomy, preparation of the supratruncal aortic segment through the lesser omentum and of the infratruncal aortic segment through a Mattox manoeuvre followed. Supratruncal clamping and opening of the aneurysm sack enabled a transection of the endovascular reconstruction and retrieval of the cranial components including the renal stent grafts. Afterwards, local thromboendarterectomy (TEA), debridement, and aneurysmorrhaphy from the coeliac trunk to the right renal artery were performed. To minimise visceral ischaemia, supratruncal clamping (15 minutes) was then switched to transrenal clamping (ischaemia of the right renal artery). Proximal anastomosis (xenograft to aorta) was completed. Since local TEA sufficed and guaranteed patent and undissected renal arteries, reconstruction of the latter was not necessary. The preliminary prepared venous bypasses of the composite graft were shortened and ligated. In continuation, infrarenal aortobi-iliac replacement was completed (transrenal clamping time: 40 minutes). After extensive irrigation of the aneurysm sac and positioning of Jackson–Pratt drainage, a transmesocolic omentoplasty was performed. The 8 hour operation was completed by temporary abdominal closure with negative pressure therapy (VAC).

Due to the long operation time, pre-established anti-aggregation, perifocal tissue inflammation, large surgical site, and systemic inflammatory response syndrome with coagulation disorder, capillary leak, and shock, massive transfusion (11 red cell and one thrombocyte concentrates) as well as administration of coagulation products were necessary. In addition to the high post-operative catecholamine requirement, renal function declined (AKIN 3). Recovery was slow during the consecutive 22 intensive care unit days. Kidney replacement therapy was not necessary as kidney function recovered. During several uncomplicated second look operations, definitive abdominal closure was achieved.

Peri-operative antibiotic therapy consisted of flucloxacillin for *S. aureus* bacteraemia. Intra-operative samples (aneurysm thrombus, explanted endografts, and aortic wall) turned positive for *S. aureus* and *Escherichia coli*, and antibiotic therapy was switched to ceftriaxone. Absence of signs of persistent infection during recovery led to a de-escalation of vancomycin and cefepime. At discharge, therapy consisted of a combination of ciprofloxacin and rifampicin.

After a total of 48 days at the centre, the patient was discharged to a rehabilitation clinic. Three month follow up revealed normal white blood cell count and CRP, absence of infection signs in the history, and clinical evaluation as well as satisfactory imaging findings. The antibiotics were discontinued, and close clinical and laboratory monitoring by the general practitioner accompanied by remote file consultation by the case leading vascular surgeon were established. A recent four year follow up showed a patent aortic reconstruction as well as freedom from infection. Dilation of the biosynthetic to common iliac artery anastomosis site on both sites, first documented one year after reconstruction, remained unchanged (Fig. 2B).

## Discussion

Open surgical conversion operations due to FB-EVAR infection are difficult and not subject to any standard of care, despite EI treatment recommendations. This case report is one of the few describing infected FB-EVAR explantation.

In the present case, major therapeutic decisions were made in a multidisciplinary setting with vascular surgeons, radiologists, and infectiologists.^3^^,^^5^

In the present case, transperitoneal total FB-EVAR removal seemed the only viable curative approach. Alternative approaches included partial FB-EVAR removal, semi-conservative treatment with drainage and irrigation, or extra-anatomic (temporary) reconstruction. These alternatives did not appear to be optimal because they either left behind infected graft components, required further surgical steps until definitive orthotopic reconstruction or have been associated with higher mortality.^3^ In particular, the option of strict conservative treatment must be considered equivalent to palliation in view of the extent of infected tissue and grafts present. According to the current recommendations, extensive thorough debridement of tissue adjacent to the graft as well as the aortic wall was performed.^5^^,^^8^

To minimise the visceral and renal ischaemia times, suprarenal clamping was only performed for proximal graft removal and sectional debridement. Afterwards, transrenal clamping was sufficient for completion of the extensive debridement of the aneurysmal sac, infected aortic wall, and retroperitoneal tissue, leaving a macroscopically clean aortic bed for *in situ* reconstruction.

The latter consisted of autologous veins and a combined xeno/biosynthetic graft. Alternative materials consist mainly of cryopreserved allografts and rifampicin bonded or silver coated synthetic grafts. The first were not available at the centre and have been associated with graft degradation,^8^ and the latter seemed inferior with respect to infection resistance compared with the above chosen materials. Today, the standard approach in infrarenal septic aortic surgery consists of *in situ* reconstruction of a factory pre-sewn bovine xenograft bifurcated prosthesis, which was not available at the time.

Finally, in accordance with guideline recommendations,^8^ transmesocolic omentoplasty was performed to cover the entire reconstruction, providing additional protection.

Precision antibiotic treatment, choice of substance, as well as duration of therapy were decided among the interdisciplinary team with the consulting infectiologists. The suspected pathogenesis of EI in the present case seemed to be a percutaneous pathogen spread, which probably took place during the aforementioned emergency coronary interventions. Indeed, one of the most frequent risk factors for EI are recent (past twelve months) vascular interventions. In contrast to the case of vascular surgical intraluminal stent graft implantation, routine antibiotic prophylaxis before coronary angiography is usually given only in immunosuppressed patients.

In the present case, it is particularly evident that the planning and implementation of treatment for aortic graft infections must be carried out in a maximum care setting. Vascular surgery skills, including subdiaphragmatic and intrathoracic reconstructions, are essential, as is the accompanying anaesthetic and intensive medical care. In addition to conventional microbial culture, on site microbiological diagnostics must also be able to offer polymerase chain reaction and sonication analyses to enable maximum germ identification.

## Conclusion

The present case demonstrates the complexity of the necessary treatment, where not only advanced surgical skills but also state of the art peri-operative care (anaesthesia, intensive care, infectiology) are crucial for patient survival. This confirms current guideline recommendations that EI should be referred for treatment to specialised vascular surgery centres.^8^ Continuous infectiological co-assessment is essential, as is close follow up.

## Funding

None.

## Conflict of interest

None.
